# N-terminal Trimming Supports Proteostasis Under Secretory Protein Mislocalization Stress

**DOI:** 10.64898/2026.10.13.756067

**Published:** 2026-10-15

**Authors:** Jasmine P Castellanos, Khanh K. Nguyen, Qiqi Lin, Ziqi Lyu, Zi Yang, Fabella Sarwono, Jean Carlo Gonzalez, Walter Nguyen, Lyna T. Huynh, Carolina Canizal, Prasanna Satpute-Krishnan, Wei Shi, Joseph C. Genereux

## Abstract

Secretory proteins that fail to enter the endoplasmic reticulum (ER) are mislocalized to the cytosol, where they threaten cellular proteostasis. How cells manage these mislocalized proteins remains poorly understood. Here, we show that inefficient Sec61 translocon activity induces a transcriptional stress response, which is enhanced by blocking NRF-1-dependent adaptation of proteasomal capacity. To maintain proteasomal capacity, thermodynamically stable mislocalized proteins persist in the cytosol of HepG2 and HEK293T cells, while mislocalized proteins that are inherently more aggregation-prone are preferentially ubiquitinated and degraded. This triaging process is accomplished through N-terminal trimming of the signal sequence of thermodynamically stable proteins, which simultaneously reduces their aggregation and prevents proteasomal degradation. These findings reveal a stability-dependent cytosolic triaging mechanism that matches substrate burden to proteasome capacity when ER import is challenged.

**TOC Graphic:**

